# Improving the Effect and Efficiency of FMD Control by Enlarging Protection or Surveillance Zones

**DOI:** 10.3389/fvets.2015.00070

**Published:** 2015-12-02

**Authors:** Tariq Halasa, Nils Toft, Anette Boklund

**Affiliations:** ^1^Section of Epidemiology, National Veterinary Institute, Technical University of Denmark, Copenhagen, Denmark

**Keywords:** foot-and-mouth disease, control, simulation model, surveillance

## Abstract

An epidemic of foot-and-mouth disease (FMD) in a FMD-free country with large exports of livestock and livestock products would result in profound economic damage. This could be reduced by rapid and efficient control of the disease spread. The objectives of this study were to estimate the economic impact of a hypothetical FMD outbreak in Denmark based on changes to the economic assumptions of the model, and to investigate whether the control of an FMD epidemic can be improved by combining the enlargement of protection or surveillance zones with pre-emptive depopulation or emergency vaccination. The stochastic spatial simulation model DTU-DADS was used to simulate the spread of FMD in Denmark. The control strategies were the basic EU and Danish strategy, pre-emptive depopulation, suppressive or protective vaccination, enlarging protection or surveillance zones, and a combination of pre-emptive depopulation or emergency vaccination with enlarged protection or surveillance zones. Herds are detected either based on basic detection through the appearance of clinical signs, or as a result of surveillance in the control zones. The economic analyses consisted of direct costs and export losses. Sensitivity analysis was performed on uncertain and potentially influential input parameters. Enlarging the surveillance zones from 10 to 15 km, combined with pre-emptive depopulation over a 1-km radius around detected herds resulted in the lowest total costs. This was still the case even when the different input parameters were changed in the sensitivity analysis. Changing the resources for clinical surveillance did not affect the epidemic consequences. In conclusion, an FMD epidemic in Denmark would have a larger economic impact on the agricultural sector than previously anticipated. Furthermore, the control of a potential FMD outbreak in Denmark may be improved by combining pre-emptive depopulation with an enlarged protection or surveillance zone.

## Introduction

Foot-and-mouth disease (FMD) is a highly contagious viral disease affecting ruminants and pigs (1–3), and an epidemic may have a large economic impact on FMD-free countries and regions (4, 5). In an FMD-free country with large exports of livestock and livestock products, it is likely that the relevance of an outbreak would not be restricted to the domestic market of the affected country, but it might also affect the international market (6). Trade restrictions would be imposed to minimize the risk of disease spread to other countries (6). The OIE allows affected countries to resume exports 3 months after the destruction of the last infected or vaccinated herd (7). However, if protective vaccination is used, export cannot be resumed until 6 months after the end of the campaign (7), and not before all vaccinated animals are tested and confirmed negative for the virus (7). The European Union (EU), on the other hand, allows affected countries to export to other member states from regions confirmed to be free of the disease (8). Nevertheless, it is expected that products will be sold with a price reduction, in order to prevent extra storage challenges and costs. Furthermore, a short export ban on all livestock products would be imposed at the beginning of the outbreak to prevent disease spread to other member states. During the 2007 UK epidemic, the EU imposed restrictions on exports of livestock and livestock products from the UK to other member states (9).

Simulation modeling of FMD is widely used as a decision support tool in contingency planning for FMD awareness and preparedness (10–14). During the 2001 UK outbreak, simulation models were used to help the veterinary authorities control the spread of the disease (15–17).

Earlier work has predicted that pre-emptive culling would often be the optimal strategy to control a hypothetical FMD outbreak in Denmark, given the assumptions (13). Suppressive vaccination (vaccinated animals are culled) resulted in slightly higher total costs than pre-emptive depopulation. Both strategies are comprised the slaughter of a large number of healthy animals. Protective vaccination, on the other hand, does not involve slaughtering healthy animals and was predicted to give the shortest epidemic duration and the lowest number of affected herds. However, the largest economic damage was predicted to follow protective vaccination due to export losses (13). More recent work has shown that enlarging the protection zone from 3 to 5 km or the surveillance zone from 10 to 15 km has a good potential to control FMD (18). However, the model included optimistic assumptions regarding exports of Danish products to the EU markets, which could have hidden the true magnitude of the positive impact of zone enlargement. The authors recommended investigating the impact of combining enlarged zones with pre-emptive culling or emergency vaccination, taking into account realistic assumptions regarding exports of Danish livestock products (18). To the best of our knowledge, the impact of combining pre-emptive depopulation or emergency vaccination with enlarged protection or surveillance zones on the epidemiological and economic consequences of an FMD epidemic has not been investigated before. This information can be used to inform decision-makers on optimal control strategies for potential FMD outbreaks in EU member states.

The objectives of this study were to estimate the economic impact of a hypothetical FMD outbreak in Denmark based on changes to the economic assumptions of the DTU-DADS model, and to investigate the epidemiological and economic impact of strategies combining pre-emptive depopulation or emergency vaccination with enlargement of the protection or the surveillance zones on the control of a hypothetical epidemic.

## Materials and Methods

### Study Population

Information on Danish herds constituted the core of the model. We used data from the Danish Central Husbandry Register (CHR), extracted for the period 1st October 2006 to 30th September 2007, as previously explained (13), in order to compare the results of this study to our previous findings (13). The data included 23,550 cattle herds, 11,473 swineherds, and 15,830 sheep and goatherds. For each herd, data included a unique identity number (CHR number), herd type, UTM geo-coordinates, number of animals, and rate of animal movements from the herd per day. Herds were grouped into three types: cattle, swine, and sheep and goats. Cattle herds were categorized as dairy or non-dairy herds. Swine herds were categorized into 19 different types based on their production type and specific pathogen-free^1^ (SPF) status (19). Sheep and goats were grouped and treated equally (referred to as sheep herds throughout the paper), since Denmark has a limited number of goat herds, and the disease dynamics are expected to be similar to those of sheep herds. When a farm included several animal species, each species was treated as a separate herd in the model and was given a separate identification number, but registered on the same location and with the same CHR number. Information about markets was also available, including the UTM geo-coordinates.

The number of animal movements per herd was summarized over 1 year, using data from the movement database between October 2006 and September 2007. The total count of movements of each animal type (cattle, weaners, sows, and sheep) for each individual herd, divided by the days counted (365), was used as lambda (λ) in a Poisson distribution simulating the movements of the individual herd. Each movement reflected a batch of animals, but without defining the number of animal within the batch. The distance for movements was calculated from the movement databases for each movement type as the Euclidean distance. One distribution of distances was used for each movement type. We simulated five different types of direct movements of animals (cattle, weaners, sows, sheep/goats, and movements to/from Bornholm^2^) and two types of market movements (to and from markets). For each type of herd, the probability of sending animals to other types of herds was calculated based on animal movement data. These probabilities were used to select the receiving herds for each simulated movement of animals. One batch of animals was regarded as one movement.

The input parameters of the model were based on Danish data; the literature and personal communication with experts are described in detail in earlier publications (13, 18) and in Table S1 in Supplementary Material.

### The Simulation Model

The model simulated the hypothetical spread of FMD between herds in Denmark using the dynamic spatial simulation model DTU-DADS (version 0.150) that runs in the freeware R (20). The model is an upgrade from DTU-DADS (version 0.14) (18), which included updating the economic assumptions that simulates export losses and updating detection parameters following re-evaluation by experts as shown in Table S2 in Supplementary Material. The major processes within the model are disease spread and detection, implementation of control strategies, which must satisfy the EU and national legislations, implementation of optional control strategies (including pre-emptive depopulation and emergency vaccination), culling of detected herds. Disease spread and control are explained in the subsequent sections. Culling, vaccination, and clinical surveillance are all based on the available resources. For example, herds to be culled are set in a queuing system and are culled when resources are available. Vaccination and clinical surveillance are implemented in a similar manner. Herds are detected either based on basic detection through the appearance of clinical signs or as a result of surveillance in the control zones.

### Disease Spread

The simulation starts in one herd (the index herd). Other studies have shown that the index herd influences the size and duration of the epidemic (19, 21). In order to include the variation caused by different index herds, we randomly selected 1,000 cattle herds as index herds. Each index herd was run in 1 iteration, resulting in 1,000 iterations per model run (scenario).

The spread of infection between herds was simulated through seven spread mechanisms: (1) direct animal movement between herds; (2) abattoir trucks; (3) milk tankers; (4) veterinarians, artificial inseminators, and/or milk controllers (medium-risk contact); (5) visitors, feedstuff, and/or rendering trucks (low-risk contact); (6) markets, and (7) local spread. Movements of live animals and animals for slaughter were simulated as probabilities for the individual herd. Based on actual movement data, a probability of movements per day was calculated for each herd. The individual daily movement rate was used as λ in a Poisson distribution to represent the number of movements per day. Similarly, the probability of abattoir deliveries per day was calculated based on actual data from herds, and used in a Poisson distribution to simulate the number of movements to the abattoir per day from the infectious herd. Thereafter, the number of herds visited by an abattoir truck on the way to the abattoir following a visit to an infected herd was estimated from a Poisson distribution with a λ dependent on the herd type. Abattoir trucks collect animals of the same species. The collection of milk, as well as medium- and low-risk contacts were simulated for the different herd types, each described by a λ in a Poisson distribution (13). Since markets in Denmark are restricted to cattle only, an infection spreading from a market can initially only affect cattle herds. The spread via markets would occur from the direct movement of infected animals to susceptible herds, or via people and vehicles that had been in contact with the infected animals.

Local spread was defined as infection due to unexplained reasons potentially contributing to the spread of disease within short distances, such as limited airborne spread, rodents, birds, and flies. Local spread was simulated as a small probability of infecting neighboring herds within a 3-km radius around the infected herds (11, 13). The 3-km radius was based on Gibbens et al. (22). Herds located on the same farm had a 95% daily chance of infection when one herd was infected (13).

The period from when a herd started to show clinical signs until it was detected was dependent on the herd type. For example, cattle herds were detected faster than sheep herds, because some sheep do not show clinical signs.

### Basic Control Strategy

Following detection of the first infected herd, a set of control measures was applied, representing the basic scenario (EU and Danish control regulations). These included (1) depopulation, cleaning, and disinfection of detected herds; (2) a 3-day national standstill on animal movements in the country; (3) implementation of a 3-km protection zone and a 10-km surveillance zone around the detected herds, in which movements between herds as well as movements in and out of the zones were restricted and herds were surveyed at least one (surveillance zone) or two (protection zone) times before lifting the restrictions; and (4) backward and forward tracing of contacts to and from detected herds. Herds that had received animals from a detected herd were also depopulated and disinfected, and in cases involving other kinds of contacts, the herds were surveyed. When a herd was subject to surveillance, the animals were inspected for clinical signs of FMD, and sheep herds were serologically tested. The daily surveillance capacity was set to 450 herds (18).

The daily depopulation capacity was set at 2,400 ruminants and 4,800 pigs. These numbers were calculated based on the number of people available for culling, the time needed to cull and the number of animals in the herds. Further details are given in the Supplementary Material of Boklund et al. (13). Detected herds had higher priority for depopulation than traced herds. If several herds were located on the same farm, all herds on the farm were depopulated when one herd was detected.

### Alternative Control Strategies

We investigated the effect of seven different control strategies, described as different scenarios. Extra control measures were always applied on top of the basic scenario. The scenarios were (1) the basic scenario (as previously described); (2) pre-emptive depopulation, including depopulation of herds within a 1-km radius around detected herds; (3) suppressive vaccination,^3^ including emergency vaccination of herds within a 1- or 2-km radius around detected herds; (4) protective vaccination,^4^ including emergency vaccination of herds within a 1- or 2-km radius around detected herds; (5) enlargement of the protection zone, from 3 to 5 km; (6) enlargement of the surveillance zone from 10 to 15 or 20 km, and finally (7) combined strategies, including pre-emptive depopulation, suppressive, or protective emergency vaccination combined with either enlarging the protection zone from 3 to 5 km or enlarging the surveillance zone from 10 to 15 km. Vaccination and depopulation were initiated after the detection of 10 infected herds, or after 14 days following the first detection of an infected herd, as recommended by the Danish Veterinary Authorities. The emergency vaccination scenario with the radius resulting in the lowest total costs was used in the scenarios where emergency vaccination was combined with enlarged protection or surveillance zones. The daily animal vaccination capacity was assumed to be 50,000 ruminants and 60,000 pigs (13). Before vaccination, cattle and pig herds were clinically surveyed and sheep herds were also serologically surveyed.

### Costs and Losses

The costs and losses of the epidemics were calculated, as presented previously (13). Briefly, the direct costs were related to surveillance, depopulation, cleaning and disinfection, empty housing, compensation, and national standstill (e.g., losses incurred by feed and transport companies and slaughter houses). The indirect costs included losses incurred from restrictions on exports to EU and non-EU countries (export loss). The method of calculating the direct costs was not changed in the current analysis. However, the calculation of indirect costs was changed by adding: (1) a complete export ban (block) on Danish exports of swine products and beef to the EU market for 14–28 days. It was assumed that storage capacity would be available for approximately 7–14 days of production, and it was therefore assumed that the loss would be equivalent to 1–21 days of production. A program evaluation and review technique (PERT) distribution with a minimum of 1, a mode of 14, and a maximum of 21 days was used to represent this lost export to EU member states. In addition, (2) a price reduction for pork and beef sold in the EU market from uninfected areas. A PERT distribution with a minimum of 5%, a mode of 6%, and a maximum of 10% was used to represent the price reduction. Total costs were calculated per iteration, and their summaries were thereafter estimated based on the 1,000 iterations.

### Sensitivity Analysis

The sensitivity of model results toward input parameters was investigated. The influence of local spread, low-risk contacts, and basic detection was examined, as these parameters were previously shown to have a major impact on our model prediction (13). The input parameters were increased or decreased by 25%, using different control scenarios. Furthermore, we investigated the influence of an increase from 25 to 50% in the price reduction on exports of pork and beef to non-EU countries, and a prolonged time to regain the free status from 3 to 6 months, though only in the basic scenario. Additionally, resources for clinical surveillance were changed from 450 to 300 or 600 herds per day only for the basic scenario, in order to study the epidemic consequences. For the sensitivity analysis, all parameters kept unchanged, but the one which was explored.

### Statistical Analysis

For all scenarios, the epidemiological and economic results were compared to the basic as well as to the optimal scenario. The optimal scenario was considered to be the scenario with the lowest total costs of the epidemics. The epidemiological results included duration of epidemics, and the numbers of infected, depopulated, and vaccinated herds, whereas economic results included the total costs and losses. To test the statistical differences between the scenarios, we used the Wilcoxon rank sum test, run in the statistical software R (20). A *p*-value threshold of <0.05 was used to determine statistical significance.

## Results

### Basic Control Strategy

The median duration of an epidemic in cattle herds was 67 days, with 17 and 185 days as 5th and 95th percentiles, respectively (Table 1). They resulted in 86 (13–368) infected herds, leading to total costs of €1,087 (768–1,766) million (Table 1).

**Table 1:** **Epidemiological and economic consequences of simulated FMD epidemics in Denmark under different control scenarios, when epidemics were initiated cattle herds**.

Scenario	Epidemic duration	Infected herds	Depopulated herds	Vaccinated herds	Total costs (€ × 10^6^)
Basic	67 (17–185)	86 (13–368)	86 (13–367)	–	1,087 (768–1,766)
PZ5	67 (18–184)^a^	81 (11–373)^a^	81 (11–372)^a^	–	1,089 (770–1,754)^a^
SZ15	59 (18–156)	74 (12–314)	74 (12–311)	–	1,052 (773–1,807)^a^
Dep14D	42 (16–78)	60 (12–184)	151 (17–509)	–	937 (761–1,187)
Dep10H	35 (15–75)	49 (12–143)	167 (20–542)	–	888 (752–1,135)
VTK14D-1 km	51 (17–104)	69 (12–233)	69 (12–231)	124 (5–481)	996 (767–1,337)
VTK10H-1 km	47 (19–97)	60 (13–188)	60 (13–187)	157 (10–553)	972 (769–1,285)
VTK14D-2 km	46 (17–88)	63 (12–207)	63 (12–204)	335 (19–1,219)	981 (768–1,299)
VTK10H-2 km	41 (19–81)	53 (13–170)	53 (13–168)	410 (40–1,432)	941 (776–1,233)
VTL14D-1 km	51 (18–100)	69 (12–221)	69 (12–219)	122 (6–463)	1,159 (920–1,475)
VTL10H-1 km	49 (19–97)	62 (13–202)	62 (12–200)	161 (11–578)	1,129 (928–1,455)
VTL14D-2 km	44 (17–79)	60 (12–188)	60 (11–184)	282 (16–1,108)	1,106 (920–1,382)
VTL10H-2 km	40 (17–75)	53 (11–160)	53 (11–158)	400 (24–1,359)	1,077 (919–1,349)^a^
Dep14D-PZ5	40 (18–78)	57 (12–176)	142 (18–471)	–	925 (763–1,185)
Dep10H-PZ5	36 (16–72)	49 (12–139)	162 (18–512)	–	888 (752–1,126)
Dep14D-SZ15	38 (18–72)	56 (12–169)	133 (18–450)	–	914 (767–1,220)
**Dep10H-SZ15**	**34 (16**–**68)**	**47 (12**–**126)**	**156 (22**–**483)**	–	**885 (755**–**1,168)**
VTK14D-2 km-PZ5	45 (18–87)	62 (12–199)	61 (12–197)	319 (23–1,137)	972 (777–1,290)
VTK10H-2 km-PZ5	42 (18–79)	55 (12–166)	54 (12–164)	427 (26–1,412)	958 (768–1,225)
VTK14D-2 km-SZ15	43 (18–78)	59 (12–184)	58 (12–183)	283 (21–1,083)	961 (775–1,371)
VTK10H-2 km-SZ15	37 (18–75)	50 (12–149)	50 (12–147)	380 (32–1,237)	935 (769–1,279)
VTL14D-2 km-PZ5	45 (18–81)	62 (12–186)	62 (12–186)	297 (19–1,041)	1,072 (907–1,316)
VTL10H-2 km-PZ5	38 (18–73)	51 (11–152)	51 (11–151)	375 (22–1,227)	1,071 (911–1,360)^a^
VTL14D-2 km-SZ15	43 (18–78)	59 (12–184)	58 (12–183)	283 (21–1,083)	1,104 (920–1,489)
VTL10H-2 km-SZ15	37 (18–75)	50 (12–149)	50 (12–147)	380 (32–1,237)	1,072 (919–1,382)^a^

*The basic scenario represents the EU and Danish control regulations (Basic), combined with the enlargement of the protection zone to 5 km (PZ5) and the surveillance zone to 15 km (SZ15), pre-emptive depopulation (Dep) over a 1-km radius around detected herds, and suppressive (VTK) or protective (VTL) emergency vaccination over a 1- or 2-km radius around detected herds. Pre-emptive depopulation and vaccination were initiated following the detection of 10 infected herds (10H) or after 14 days (14D) from the detection of the first infected herd. Scenarios combining pre-emptive depopulation over 1 km, suppressive or protective emergency vaccination over 2 km and enlarged protection or surveillances zones are also included. Results are presented as a median with 5th and 95th percentiles. The optimal scenario (lowest total cost) is in bold. All variables were statistically significantly different (*p*-value <0.05) from the corresponding variable in the basic control strategy, unless superscripted by “^a^”*.

*^a^Not significantly different from the corresponding variable in the basic scenario (*p*-value ≥0.05)*.

In certain scenarios, the alternative control strategy was initiated after the detection of at least 10 infected herds. The detection of 10 infected herds was achieved in 968 out of 1,000 iterations. These 10 herds were detected within a median value of 4 days following first detection, though this varied with 5th and 95th percentiles of 1–14 days, respectively. This indicates that the initiation of the alternative control strategy in these scenarios, would usually start 4 days following first detection, but it could start as early as 1 day following first detection or after 14 days following first detection.

### Alternative Control Strategies

An increase in the size of the protection zone from 3 to 5 km did not decrease the size, duration, and total costs of the epidemics, whereas an increase in the size of the surveillance zone from 10 to 15 km decreased the size and duration of the epidemics, but not the total costs (Table 1). All other alternative scenarios resulted in shorter epidemic duration and fewer infected herds than the basic scenario (Table 1).

Furthermore, combinations of pre-emptive depopulation with enlarged protection or surveillance zones resulted in lower total costs of the epidemics compared to the basic scenario (Table 1). The lowest total costs (optimal scenario) were estimated when pre-emptive depopulation was initiated following the detection of 10 infected herds, and combined with enlarged surveillance zones (Dep10H-SZ15). Nevertheless, there were no statistically significant differences in the total costs between this scenario and scenarios with depopulation initiated after detection of 10 infected herds (Dep10H), or depopulation initiated after detection of 10 infected herds and combined with enlargement of the protection zone (Dep10H-PZ5). Nevertheless, Dep10H-SZ15 resulted in the minimum number of infected herds and a smaller variation in epidemic duration and numbers of infected herds when compared to the other scenarios.

**Figure 1:**
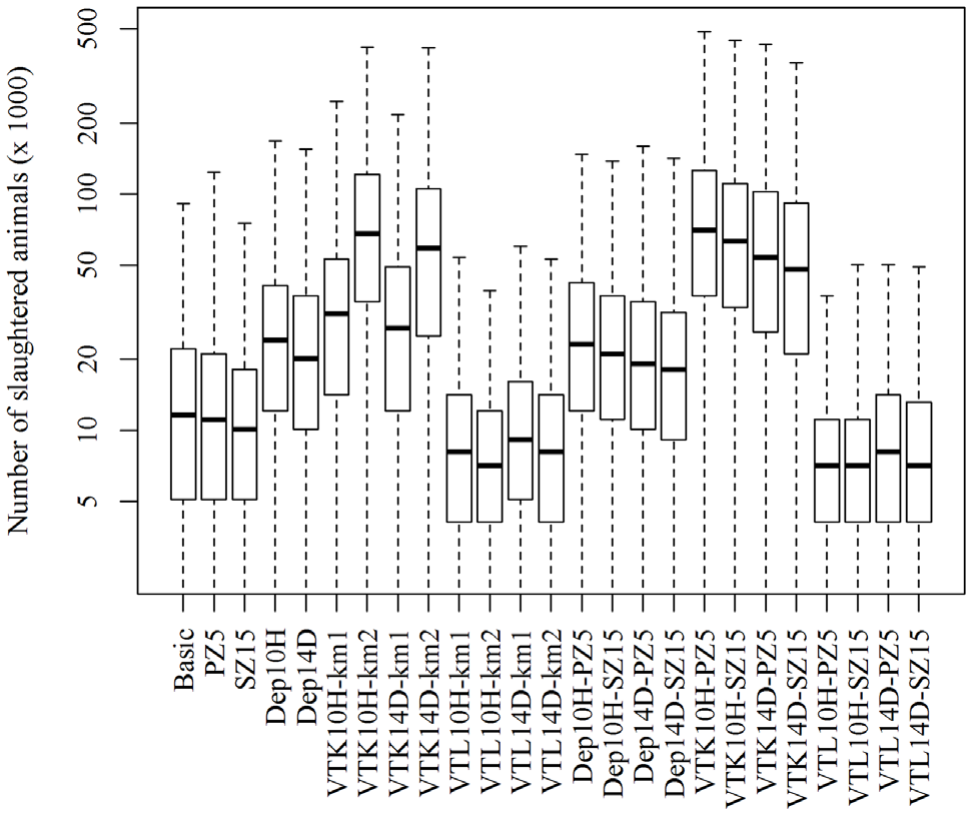
**Numbers of slaughtered animals using different strategies to control 1,000 simulated FMD epidemics in Denmark, all initiated in cattle herds**. The basic scenario represents the EU and Danish control measures (Basic), combined with the enlargement of the protection zone to 5 km (PZ5) and the surveillance zone to 15 km (SZ15), pre-emptive depopulation (Dep) over a 1-km radius around detected herds, and suppressive (VTK) or protective (VTL) emergency vaccination over a 1- or 2-km radius around detected herds. Pre-emptive depopulation and vaccination were initiated either following the detection of 10 infected herds (10H) or after 14 days (14D) from the detection of the first infected herd. Scenarios combining pre-emptive depopulation over 1 km, suppressive or protective emergency vaccination over 2 km and enlarged protection or surveillances zones are also included.

**Figure 2:**
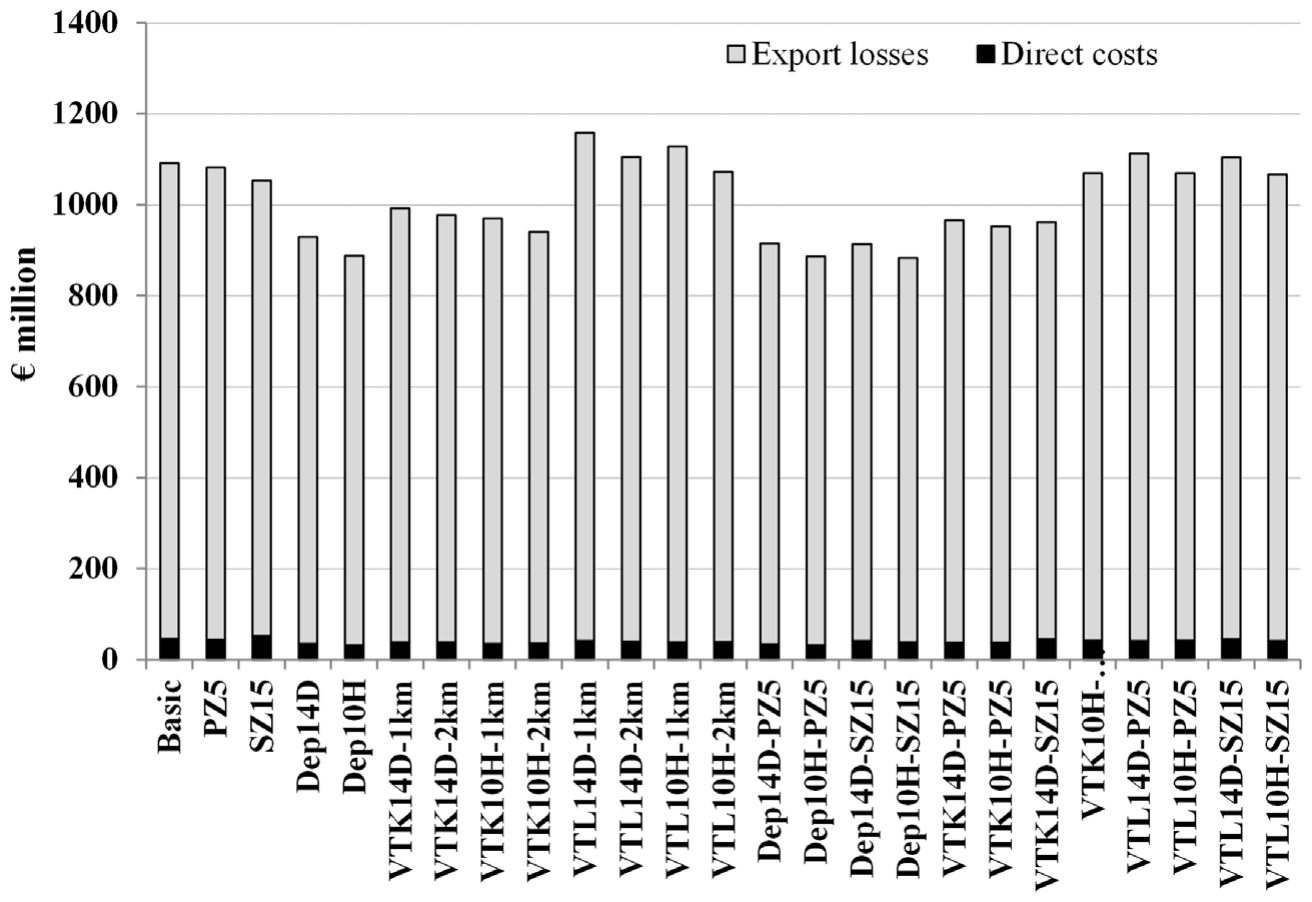
**Median direct costs and export losses using different strategies to control 1,000 simulated FMD epidemics in Denmark, all initiated in cattle herds**. The basic scenario represents the EU and Danish control measures (Basic), combined with the enlargement of the protection zone to 5 km (PZ5) and the surveillance zone to 15 km (SZ15), pre-emptive depopulation (Dep) over a 1-km radius around detected herds, and suppressive (VTK) or protective (VTL) emergency vaccination over a 1- or 2-km radius around detected herds. Pre-emptive depopulation and vaccination were initiated either following the detection of 10 infected herds (10H) or after 14 days (14D) from the detection of the first infected herd. Scenarios combining pre-emptive depopulation over 1 km, suppressive or protective emergency vaccination over 2 km and enlarged protection or surveillances zones are also included.

The scenario (Dep10H-SZ15) reduced the median epidemic duration by 33 days, the median number of infected herds by 39 herds, and the median total costs by €202 million when compared to the basic scenario (Table 1). As expected, the number of slaughtered animals increased significantly (*p*-value <0.05) in the scenarios where pre-emptive depopulation and suppressive vaccination were used, compared to the other scenarios (Figure 1). The vast majority of the economic losses were due to export losses (Figure 2).

### Sensitivity Analysis

**Figure 3:**
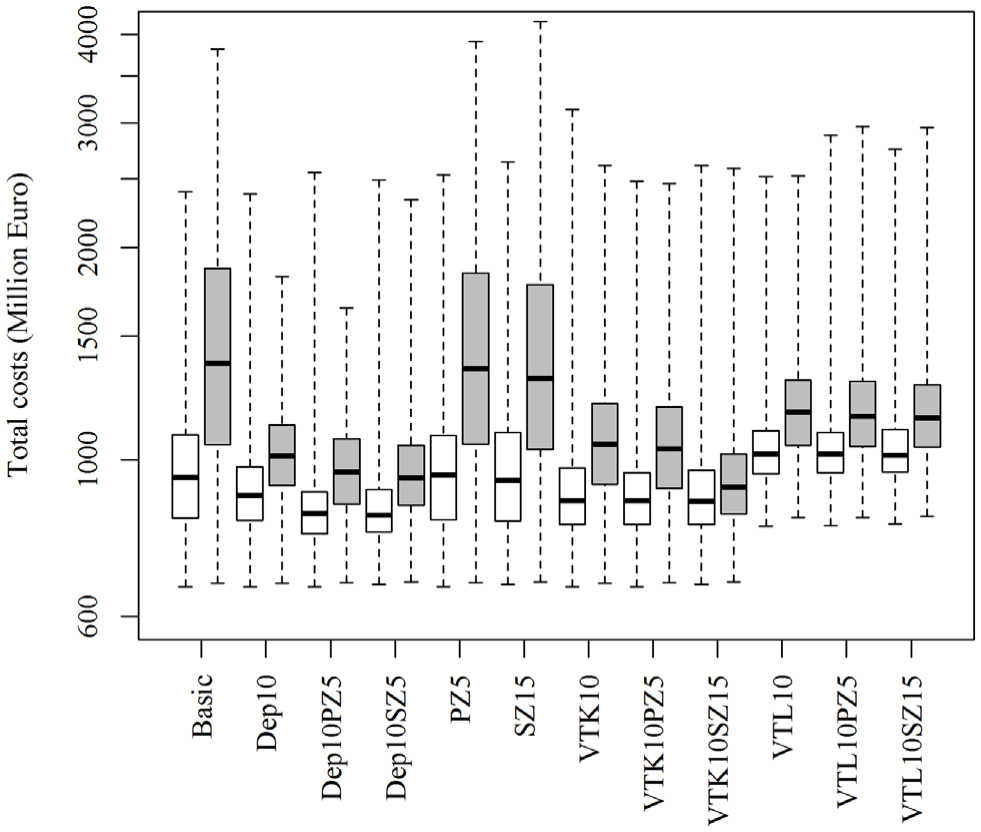
**Total costs using different strategies to control 1,000 simulated FMD epidemics in Denmark, all initiated in cattle herds, when the *probability of low-risk contacts are decreased (white boxes) or increased (gray boxes)* by 25%**. The basic scenario represents the EU and Danish control regulations (Basic), combined with the enlargement of the protection zone to 5 km (PZ5) and the surveillance zone to 15 km (SZ15), pre-emptive depopulation (Dep) over a 1-km radius around detected herds, and suppressive (VTK) or protective (VTL) emergency vaccination over a 2-km radius around detected herds. Pre-emptive depopulation and vaccination were initiated following the detection of 10 infected herds (10H). Scenarios combining pre-emptive depopulation over 1 km, suppressive or protective emergency vaccination over 2 km and enlarged protection or surveillances zones are also included.

**Figure 4:**
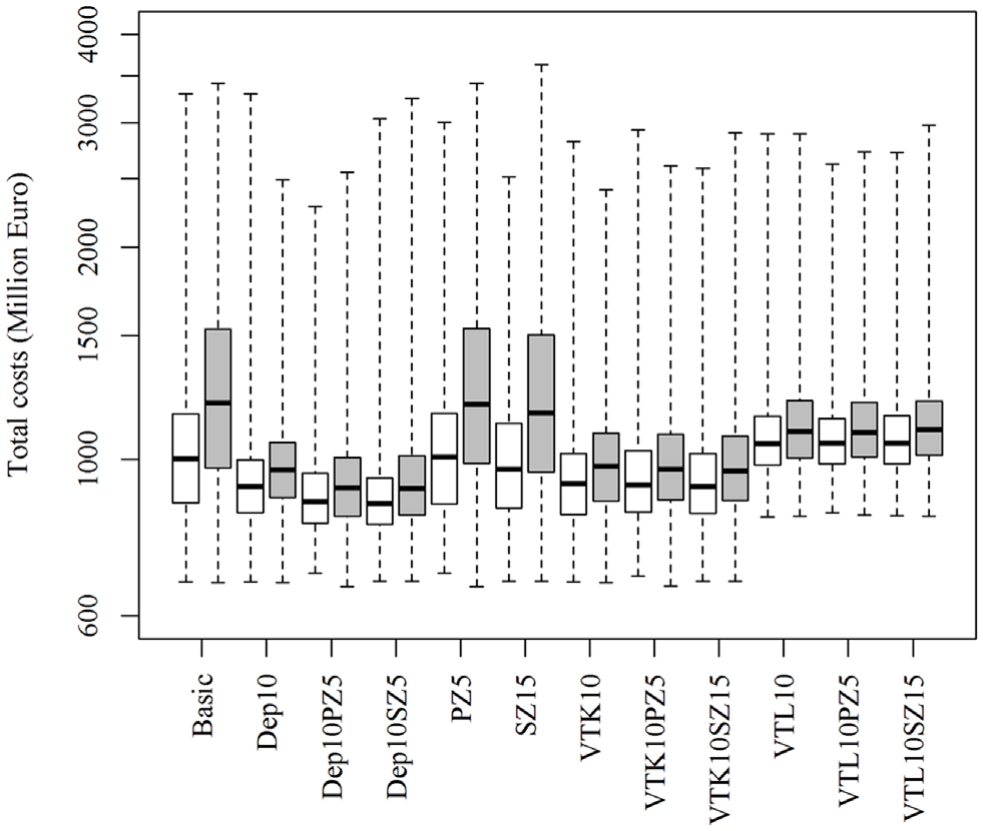
**Total costs using different strategies to control 1,000 simulated FMD epidemics in Denmark, all initiated in cattle herds, when the *probability of local spread is decreased (white boxes) or increased (gray boxes)* by 25%**. The basic scenario represents the EU and Danish control regulations (Basic), combined with the enlargement of the protection zone to 5 km (PZ5) and the surveillance zone to 15 km (SZ15), pre-emptive depopulation (Dep) over a 1-km radius around detected herds, and suppressive (VTK) or protective (VTL) emergency vaccination over a 2-km radius around detected herds. Pre-emptive depopulation and vaccination were initiated following the detection of 10 infected herds (10H). Scenarios combining pre-emptive depopulation over 1 km, suppressive or protective emergency vaccination over 2 km and enlarged protection or surveillances zones are also included.

**Figure 5:**
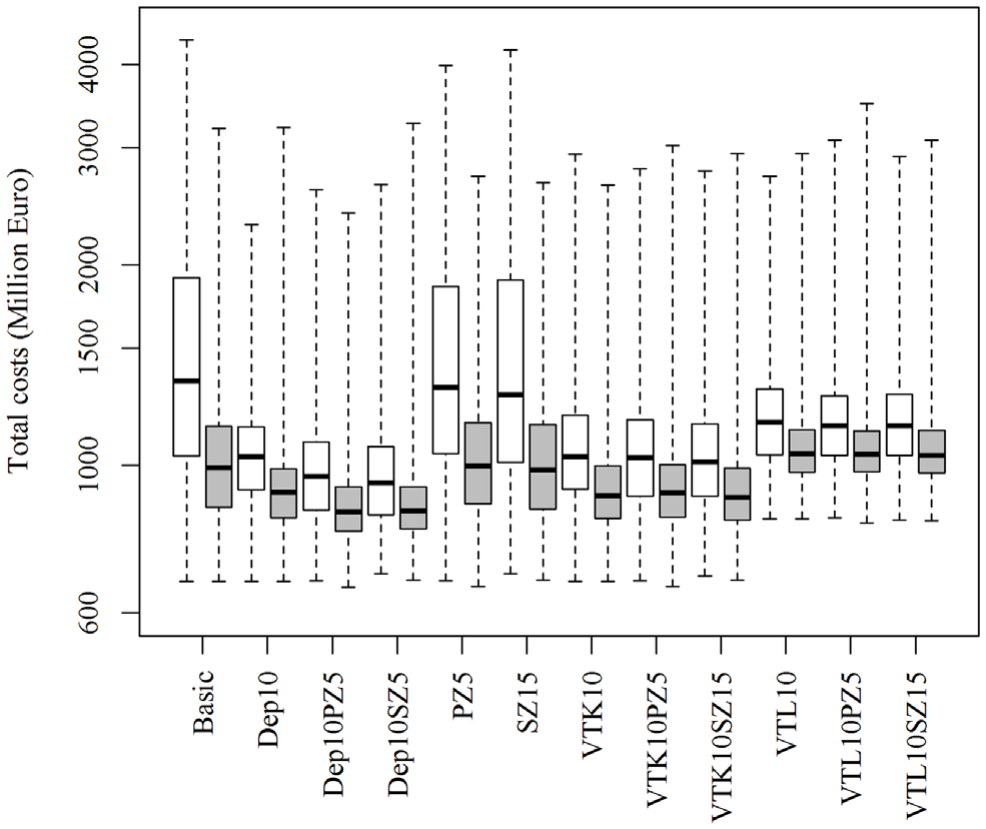
**Total costs using different strategies to control 1,000 simulated FMD epidemics in Denmark, all initiated in cattle herds, when *the probability of basic detection is decreased (white boxes) or increased (gray boxes)* by 25%**. The basic scenario represents the EU and Danish control regulations (Basic), combined with the enlargement of the protection zone to 5 km (PZ5) and the surveillance zone to 15 km (SZ15), pre-emptive depopulation (Dep) over a 1-km radius around detected herds, and suppressive (VTK) or protective (VTL) emergency vaccination over a 2-km radius around detected herds. Pre-emptive depopulation and vaccination were initiated following the detection of 10 infected herds (10H). Scenarios combining pre-emptive depopulation over 1 km, suppressive or protective emergency vaccination over 2 km and enlarged protection or surveillances zones are also included.

Increasing or decreasing the low-risk contacts has a substantial impact on the model prediction of the total costs of the epidemics for the different control scenarios (Figure 3). Similarly, changes in the probabilities of local spread and disease detection were highly influential, as shown in Figures 4 and 5, respectively. The differences in total costs between the basic scenario and the scenarios where the frequency of low-risk contacts, probabilities of local spread and disease detection were changed were statistically significant (*p*-value <0.05). Still, depopulation following the detection of 10 infected herds combined with a surveillance zone of 15 km (Dep10H-SZ15) remained the control scenario with the lowest total costs.

**Figure 6:**
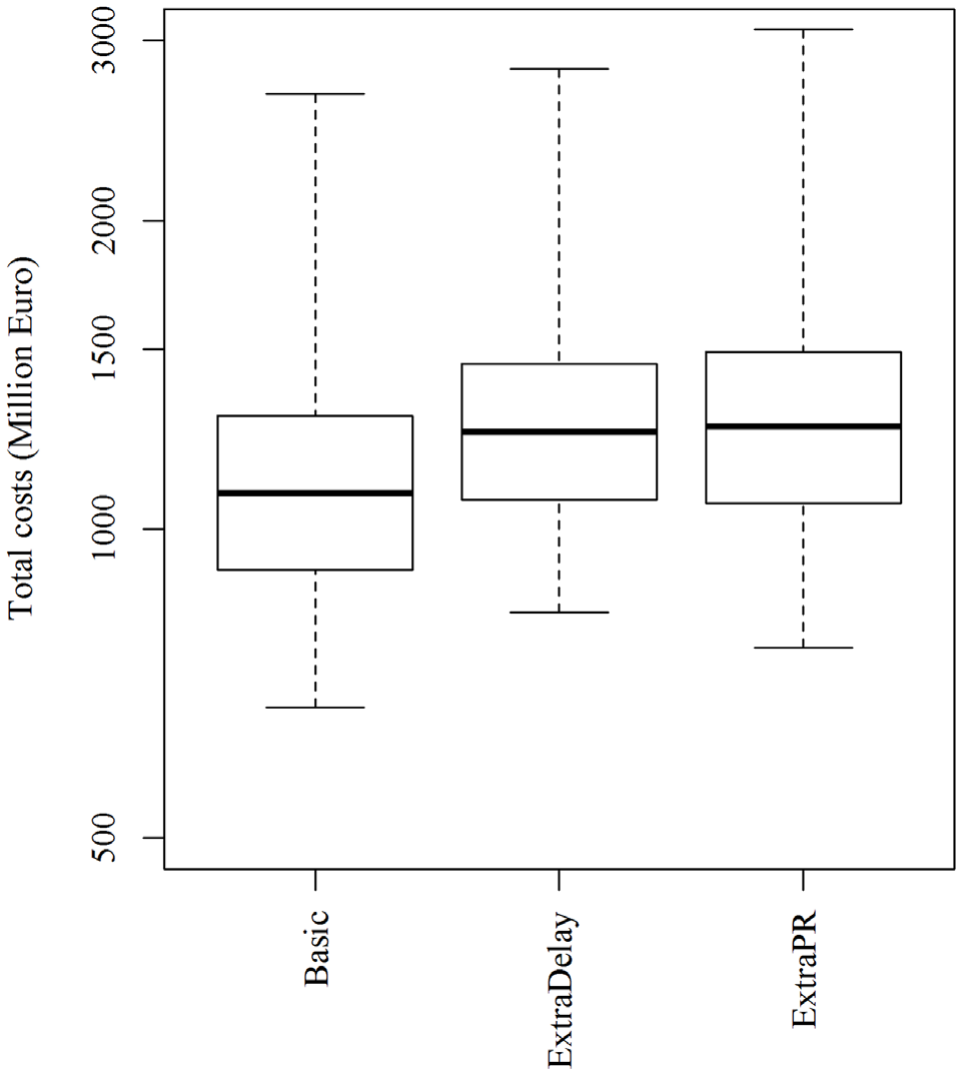
**Total costs of simulated FMD epidemics using the basic control scenario, which represents the EU and Danish control measures, when the *free status was regained 3 months (basic) or 6 months (extraDelay)* after the depopulation of the last detected herd, and when products intended for export to non-EU countries were sold in the EU market with a *price reduction of 25% (basic) or 50% (extraPR)***. Epidemics were initiated in cattle herds.

Changing the resources for clinical surveillance from 450 to 300 or 600 herds per day resulted in a marginal and statistically insignificant change in the epidemic duration, the number of infected and depopulated herds and the total costs of the epidemics (Table 2). Increasing the delay on the export of products to non-EU countries from 3 to 6 months following the depopulation of the last detected herd increased the total costs of epidemics dramatically (Figure 6). Similarly, when the export loss on products meant for export to non-EU countries, but sold in the EU market was increased from 25 to 50%, and an extra €175 million (based on median values) was lost (Figure 6).

**Table 2:** **Sensitivity analysis on resources for clinical surveillance**.

	Basic-survey 450 herds/day	Basic-survey 300 herds/day	Basic-survey 600 herds/day
Epidemic duration (days)	67 (17–185)	66 (18–192)^a^	64 (18–178)^a^
Infected herds	86 (13–368)	82 (12–375)^a^	81 (12–353)^a^
Depopulated herds	86 (13–367)	82 (12–372)^a^	81 (12–251)^a^
Total costs (€ × 10^6^)	1,087 (768–1,766)	1,084 (765–1,939)^a^	1,074 (767–1,681)^a^

*The influence on the predicted durations, number of infected and depopulated herds and total costs in simulated FMD epidemics in Denmark. Simulations were run with the basic scenario, representing the EU and Danish control regulations, and resources were changed from 450 to 300 or 600 herds per day. Epidemics were initiated in cattle herds. Results are presented as a median with 5th and 95th percentiles*.

*^a^Not significantly different from the corresponding variable in the basic scenario (*p*-value ≥0.05)*.

## Discussion

Pre-emptive depopulation following the detection of 10 infected herds, combined with enlarging the surveillance zone from 10 to 15 km (Dep10H-SZ15) resulted in the lowest total costs (Table 1). Interestingly, the total costs of the epidemics using this scenario did not significantly differ from those using pre-emptive depopulation following the detection of 10 infected herds combined with enlarging the protection zone from 3 to 5 km (Dep10H-PZ5). Nevertheless, combining the enlargement of the protection zone resulted in the smallest 95th percentile of the total costs of the epidemics (Table 1), making this scenario as an insurance against large epidemics.

Clinical surveillance within the protection and surveillance zones is useful for the early detection of infected herds in order to limit disease spread (18). Since the virus is able to spread over long distances through animal movements and indirect contacts (23), enlarging the surveillance zone is expected to limit this spread due to the restrictions on animal movements and indirect contacts, and clinical surveillance is expected to lead to earlier detection. A high herd density will lead to more local spread, and larger zones will most likely be effective in decreasing the epidemic size and duration in such areas. However, a high herd density will also result in a large number of surveyed herds, which can cause problems in situations with limited resources for surveillance. Herd density is relatively high in Denmark, but compared to other countries such as the Netherlands or certain areas in Germany (e.g., lower Saxony), the herd density is relatively low. Yet, the positive effect of enlarging the protection or surveillance zones was still observed. We therefore speculate that enlarging these zones may also have a positive effect in countries with high herd density areas.

Pre-emptive depopulation has frequently been used to control FMD outbreaks (6) and has been predicted to considerably limit disease spread in other simulation studies, given the assumptions of the models (11–13, 16, 24). Enlarged surveillance zones have been predicted to reduce the epidemic duration and number of affected herds, whereas enlarged protection zones were predicted to cause minimal losses (18). The combination of pre-emptive depopulation with zone enlargement, as investigated in the current study, may be a good strategy in controlling an FMD epidemic in Denmark.

Initiating pre-emptive depopulation following the detection of 10 infected herds was frequently predicted to be more profitable than waiting 14 days following the first detection, since 10 infected herds were often detected within this timeframe (see Basic Control Strategy under the Section “Results”), and thus disease spread would be controlled earlier than 14 days. This timeframe of 14 days was chosen following the discussion with the veterinary authorities, where it was noted that extra control measures (especially emergency vaccination) would, if necessary, only be implemented 14 days following the detection of the first epidemic, as previously indicated (23).

Emergency vaccination was used to control the spread of FMD in the Netherlands in 2001 (21). The currently available FMD vaccines do not provide complete protection and require a number of days before immunity is built (25), making pre-emptive depopulation a cheaper choice than emergency vaccination (Table 1). Furthermore, the delay on export when protective emergency vaccination is applied is two times longer than the delay when pre-emptive depopulation or suppressive vaccination is applied. This will result in extra losses for countries with large exports, which would disqualify protective vaccination as a feasible control strategy for FMD when compared to the other strategies. Nevertheless, this strategy prevents the mass slaughter of a large number of animals (Figure 1).

Previous work (24) has shown that a radius of 40 km would be the optimum vaccination radius in Denmark. In the current study, the implemented zones for pre-emptive depopulation and vaccination were chosen following thorough discussions with the veterinary authorities and the industry. Due to the relatively high density of herds in the country, larger zones would result in a substantial number of animals to be culled in the case of pre-emptive depopulation and suppressive vaccination, and in very large areas restricted from exporting, as well as a considerable number of herds included in the surveillance zones. Therefore, the authors, in agreement with experts from the Veterinary and Food Administration and from the industry, did not find the use of large zones as suggested by Tildesley and Keeling to be realistic in Denmark.

Neither an increase nor a decrease in the surveillance capacity significantly affects the total costs of the epidemics (Table 2). However, when we compare the 95th percentiles of the basic scenario and the scenario with reduced resources, there appears to be an effect of reduced surveillance capacity. This is consistent with previous findings, which showed that available resources for clinical surveillance in Denmark are generally sufficient to fulfill the regulations, but that reducing the resources might (in the extremes) result in large economic damage (18).

As previously shown (13), export losses are the driving force of the total costs of the simulated Danish epidemics (Figure 2). We included a complete block on export of swine products and beef to the EU market after the first confirmation of FMD as a new feature in the economic analysis, compared to earlier work (13). During the 2007 UK FMD epidemic, the EU prohibited exports of live animals and meat products from the UK, in order to prevent disease spread to other member states (9). Approximately 70% of the total exports of Danish swine products are exported to the EU market (26); hence, an epidemic in Denmark might be a risk for other member states. Recent work has in fact shown that given the livestock-related contact patterns between Denmark and other EU member states, an epidemic of African swine fever in Denmark would have a high probability of spreading to other member states (27). The assumption regarding an EU block on Danish exports was closely discussed with the veterinary authorities and the industry, and an export loss to the EU market for a short period was considered to be realistic. This period would include the necessary time until disease spread is contained, or free regions are demarked.

Furthermore, we assumed that products exported to the EU market from free regions (after the EU export block) would be sold with a price reduction varying between 5 and 10%, with 6% as the most likely value. The price reduction is based on the extra supply caused by exports intended for the non-EU market being sold in the EU market. This extra supply would affect the general price level of swine products. Furthermore, each different swine product is normally sold on the market that pays the highest price for the product. The limited access to these special markets during an FMD outbreak would result in a general reduction in the price of these products. In addition, during an FMD outbreak, the Danish industry would want to ensure the sale of its products, due to the country’s large swine production limiting the storage capacity. Therefore, lowering the prices is expected to facilitate the sale of the products.

As previously mentioned, the model assumes that products intended for export to non-EU markets would be sold in the EU market with a price reduction of 25%. In order to make the sale of these products attractive in the EU market, a high price reduction of 25% may be necessary. A higher price reduction (50%) would result in extra losses (Figure 6). This was also the case when the delay in regaining the free status was increased from 3 to 6 months (Figure 6).

Although these assumptions have been thoroughly discussed with the veterinary authorities and the industry, it is almost impossible to predict the reaction of the markets to Danish livestock products during and after an FMD epidemic in Denmark. EU countries are obligated to follow the EU regulations, but non-EU member states might not obey the OIE regulations, and might demand a longer time to do thorough risk analyses before re-opening their markets. Politics may also play a role in making this decision. Nevertheless, an FMD outbreak in a country with large exports of livestock and livestock products is expected to be highly detrimental to the livestock industry of that country (6).

The model prediction of the optimal control strategy seems to be consistent, whether influential parameters were increased or decreased (as shown in Figures 3–5), and can therefore provide robust predictions. Nevertheless, conclusions of simulation models are highly depend on the epidemiological parameters of the model and should therefore be interpreted with caution.

The results of the current study complement our previous work (13) and show that the economic impact of an FMD outbreak in Denmark may be much larger than initially predicted (13). An outbreak initiated in cattle herds and controlled using the basic control strategies was initially predicted to cost between approximately €550 and €650 million (13), but the current estimation is €1,087 million. Furthermore, our earlier work (18) showed that enlarging the protection zone could improve the control of an FMD epidemic. The current study shows that control of an FMD outbreak may be further improved by combining pre-emptive depopulation with an enlarged surveillance or protection zone. The results can be used to inform decision-makers on new methods to improve the control of FMD in countries with large exports of livestock and livestock products, such as Denmark.

## Author Contributions

TH, AB, and NT designed discussed the study. TH carried out the programming part, ran the analyses, and wrote the manuscript. AB and NT gave feedback to the manuscript.

## Conflict of Interest Statement

The authors declare that the research was conducted in the absence of any commercial or financial relationships that could be construed as a potential conflict of interest.

## Footnotes

^1^Specific pathogen free herds are regularly checked for freedom from a number of diseases according to the health declaration of the program. A herd can be free from all or some of the seven diseases included in the declaration system. The owner of the herd is obliged to inform the SPF company and buyers about the health status in the herd and to follow a certain set of rules regarding biosecurity (http://spfsus.dk/en)

^2^Bornholm is a Danish island located 150 km east of Zealand in the Baltic sea and has therefore a different movement patterns than the other parts of the country.

^3^Emergency vaccination followed by depopulation when resources are available.

^4^Emergency vaccination where vaccinated animals can be differentiated from infected animals, and where they are kept until slaughter.
