# Supplementary material for: Improving the Effect and Efficiency of FMD Control by Enlarging Protection or Surveillance Zones
Source: Front Vet Sci. 2015 Dec 2;2:70. doi: 10.3389/fvets.2015.00070 (PMC4675515; doi:10.3389/fvets.2015.00070)
Supplement: Supplementary file 1 [file Table_1.DOCX]

Supplementary Material

Improving the effect and efficiency of FMD-control by enlarging protection or surveillance zones

Tariq Halasa*, Nils Toft, Anette Boklund

*** Correspondence:** Tariq Halasa: tahbh@vet.dtu.dk

**Supplementary Table 1. Parameters, values and assumptions used to parameterize the simulation model**

| **Parameter description** | **Value** | **Reference** |  |
| --- | --- | --- | --- |
| Daily average number of animal shipments to other herds | Poisson (λ_Herd-specific_) | Movement data from Danish CHR and movement database for swine |  |
| Daily average number of animal shipments to abattoir | Poisson (λ_Herd-specific_) | Movement data from Danish CHR and movement database for swine |  |
| Daily average number of visits by a milk tank | Poisson (λ = 0.6) | Data from Danish abattoirs |  |
| Daily average medium risk contacts (vets, AI and milk controllers) | Lookup table: dairy cattle: Poisson (λ = 0.2347), non-dairy cattle: Poisson (λ = 0.0472), indoor swine: Poisson (λ = 0.036), outdoor swine: Poisson (λ = 0.0027), sheep: Poisson (λ = 0.012) | Combined of interviews with veterinarians, artificial inseminators and milk controllers |  |
| Daily average low risk contacts (nonprofessional visitors, trucks –rendering, feed, milk tankers) | Lookup table: cattle: Poisson (λ = 0.6129), indoor swine: Poisson (λ = 0.22), outdoor swine: Poisson (λ = 0.1837), sheep: Poisson (λ = 0.2065) | Combined from published data (Boklund et al., 2003/2004), expert opinion, and truck data |  |
| Daily number of cattle movements to market | Poisson (λ = 0.0014) | See calculations in Boklund et al. (2013) |  |
| Number of extra contacts from the market | Poisson (λ = 3.5) | See calculations in Boklund et al. (2013) |  |
| Probability of infection from animal movement that originated from and infectious herd | Pert (min=0.9, mode=0.95,max=1) | Based on Bates et al. (2003) |  |
| Probability of infection via medium risk contact from an infectious herd | Lookup table: cattle: pert (min=0.1, mode=0.5,max=0.9), indoor swine: pert (min=0.05, mode=0.2,max=0.9), outdoor swine: pert (min=0.1, mode=0.35, max=0.9), sheep: pert (min=0.1, mode=0.5,max=0.9) | See calculations in Boklund et al. (2013) |  |
| Probability of infection via low risk contact from an infectious herd | Lookup table: cattle: pert (min=0.005, mode=0.175,max=0.35), indoor swine: pert (min=0.005, mode=0.1,max=0.35), outdoor swine: pert (min=0.005, mode=0.175, max=0.35), sheep: pert (min=0.005, mode=0.175,max=0.35) | See calculations in Boklund et al. (2013) |  |
| Probability of disease transmission through a milk tank | pert distribution (min=0.005, mode=0.175,max=0.35) | See calculations in Boklund et al. (2013) |  |
| Probability of disease transmission through market contact | Normal (mean = 0.415, std=0.06) | See calculations in Boklund et al. (2013) |  |
| Probability to become infected through local spread at a specific day given distance from an infectious premise | Lookup table: from 0 to 0.1 km: 0.95, from 0.1 to 1 km: 0.012, from 1 to 2 km: 0.004, from 2 to 3km: 0.001 | Based on Bates et al. (2003) |  |
| Day at which the epidemic is detected | Pert (min=18, mode=21, max=23) | See Halasa and Boklund (2014) |  |
| Capacity for depopulation per day | Cattle and sheep: 2000 animals  swine: 4800 animals | Expert opinion based on experts from The Danish Veterinary and Food Administration and from The Danish Agriculture and Food Council | |
| Capacity for vaccination per day | Cattle and sheep: 60000 animals  swine: 50000 animals | Expert opinion based on experts from The Danish Veterinary and Food Administration and from The Danish Agriculture and Food Council | |
| Capacity for surveillance per day | 450 herds | See Halasa and Boklund (2014) | |
| Probability of imposing a successful restrictions on animal movements within the protection and surveillance zones | Pert (min=0.95, mode=0.98,max=1) | Expert opinion of The Danish Veterinary and Food Administration | |
| Probability of imposing a successful restrictions on medium risk contacts within the protection and surveillance zones | Pert (min=0.7, mode=0.8,max=0.95) | Expert opinion of The Danish Veterinary and Food Administration | |
| Probability of imposing a successful restrictions on low risk contacts within the protection and surveillance zones | Pert (min=0.2, mode=0.3,max=0.5) | Expert opinion of The Danish Veterinary and Food Administration | |
| Probability to trace animal movements | Constant = 0.98 | Expert opinion of The Danish Veterinary and Food Administration | |
| Probability of disease detection from a traced animal movement | Constant = 1 | Expert opinion of The Danish Veterinary and Food Administration | |
| Probability to trace abattoir contact and survey the herd | Constant = 0.88 | Expert opinion of The Danish Veterinary and Food Administration | |
| Probability to trace a milk tank contact and survey the herd | Constant = 0.72 | Expert opinion of The Danish Veterinary and Food Administration | |
| Probability to trace medium risk contact and survey the herd | Constant = 0.4 | Expert opinion of The Danish Veterinary and Food Administration | |
| Probability to trace low risk contact and survey the herd | Constant = 0.25 | Expert opinion of The Danish Veterinary and Food Administration | |
| Number of days required to trace animal shipments | Uniform (min=0, max=2) | Expert opinion of The Danish Veterinary and Food Administration | |
| Number of days required to trace medium risk contacts | Uniform (min=1, max=2) | Expert opinion of The Danish Veterinary and Food Administration | |
| Number of days required to trace low risk contacts | Uniform (min=0, max=4) | Expert opinion of The Danish Veterinary and Food Administration | |
