# Supplementary material for: Improving the Effect and Efficiency of FMD Control by Enlarging Protection or Surveillance Zones
Source: Front Vet Sci. 2015 Dec 2;2:70. doi: 10.3389/fvets.2015.00070 (PMC4675515; doi:10.3389/fvets.2015.00070)
Supplement: Supplementary file 2 [file Table_2.DOCX]

Supplementary Material

Improving the effect and efficiency of FMD-control by enlarging protection or surveillance zones

Tariq Halasa*, Nils Toft, Anette Boklund

*** Correspondence:** Tariq Halasa: [tahbh@vet.dtu.dk](mailto:tahbh@vet.dtu.dk)

Experts in FMD epidemiology and virology from Friedrich Loeffler Institute (FLI, Germany) re-evaluated the detection probabilities. Following re-assessment and discussions the following probabilities were proposed. Original values can be found in Boklund et al. (2013).

**Supplementary Table 2. Detection probabilities for cattle, swine and sheep herds using basic surveillance (farmers) and surveillance of traced herds and herds in control zones**

| **Day** | **Cattle and Swine (basic detection)^1^** | **Sheep (Basic detection and detection from clinical investigation)^1^** | **Cattle and swine (detection after clinical surveillance)^1^** | **Sheep (detection after serology testing) ^2^** |
| --- | --- | --- | --- | --- |
| **1** | 0.087 | 0.018 | 0.175 | 0 |
| **2** | 0.175 | 0.032 | 0.699 | 0 |
| **3** | 0.699 | 0.249 | 0.873 | 0 |
| **4** | 0.873 | 0.481 | 0.932 | 0 |
| **5** | 0.932 | 0.591 | 1 | 0 |
| **6** | 1 | 0.689 | 1 | 0 |
| **7** | 1 | 0.751 | 1 | 0 |
| **8** | 1 | 0.790 | 1 | 0 |
| **9** | 1 | 0.80 | 1 | 0.50 |
| **10** | 1 | 0.80 | 1 | 0.50 |
| **11** | 1 | 0.80 | 1 | 0.50 |
| **12** | 1 | 0.80 | 1 | 0.50 |
| **13** | 1 | 0.80 | 1 | 0.50 |
| **14** | 1 | 0.80 | 1 | 0.95 |

^1^ Probability of detection following appearance of clinical signs

^2^ Probability of detection following infection
